# SREBF1, a target gene of multiple sclerosis and coronary heart disease: based on mendelian randomization study

**DOI:** 10.1186/s41065-025-00388-6

**Published:** 2025-02-14

**Authors:** Linqin Du, Yangyang Cui, Yang Zhou, Ofe Eugene Kwaku, Xuefeng Ding, Lang Zeng, Shikang Li, Lijuan Xiong, Yonghong Zhang, Peng Zhou, Kun Wang, Rongchuan Yue

**Affiliations:** 1 Department of Cardiology, Affiliated Hospital of North Sichuan Medical College, No. 63, Wenhua Road, Nanchong, Sichuan Province 637000 P. R. China; 2 Department of Cardiology, People’s Hospital of Guang ’an District, Guang ’an, Sichuan Province 638550 P.R. China; 3 Department of Critical Care Medicine, Affiliated Hospital of North Sichuan Medical College, Nanchong, 637000 P. R. China

**Keywords:** Multiple sclerosis, Coronary heart disease, Mendelian randomization, Biomarkers, SREBF1

## Abstract

**Background and purpose:**

Research shows that people with multiple sclerosis (MS) are more likely to experience cardiovascular complications. However, the precise mechanisms underlying this association remain unclear. This study investigated the causal relationship between MS and coronary heart disease (CHD) using Mendelian randomization (MR) techniques to clarify direct effects and identify relevant target genes.

**Methods:**

We conducted various methods, including two-sample MR. method, reverse, and multivariable MR analyses, to examine the causal relationship between MS and CHD. These. methodologies effectively mitigate confounding variables and neutralize adverse causal effects. Additionally, the study explored the involvement of social factors through a two-step MR analysis. The research team performed a thorough screening of differentially expressed genes in MS based on GEO database, identifying potential target genes that may be associated with genetic risk of CHD. Enrichment analyses and protein-protein interaction studies were used to elucidate biological functions associated with these genes. We included colocalization analysis and summary data-based Mendelian randomization (SMR) method for further screening of core genes to obtain target genes.Finally, we investigated how these genes might affect health by conducting a phenome-wide MR analysis.

**Results:**

Our findings revealed that genetic predisposition to MS significantly increases the risk of CHD, with an IVW-MR analysis yielding an odds ratio of 1.091 (95% CI: 1.030, 1.155, *P* = 0.0029). Mediation analysis revealed that frailty mediated 20.2% of the effect of MS on CHD (*P* = 0.026), suggesting that frailty is a critical pathway in this relationship. Additionally, low-density lipoprotein (LDL) is associated with an increased risk of developing both MS and CHD. We identified 3025 differentially expressed genes and 130 genes causally linked to CHD. Protein-protein interaction network analysis identified 77 interacting genes, with core genes such as SREBF1 involved in organelle regulation and nucleic acid metabolism. Colocalization analysis further supported the presence of shared genetic variants between IL6R and SREBF1 associated with CHD, with posterior probabilities (PPH4) of 90.2% and 92.3%, respectively. Interestingly, summary mendelian randomization (SMR) analysis revealed that SREBF1 may be a target gene for MS(bSMR=-0.174,PSMR = 0.0218, PHEIDI = 0.2806, topSNP: rs12951376). Further analysis of the phenome-wide MR did not find significant evidence of side effect associated with targeted therapy against SREBF1.

**Conclusion:**

This study provided genetic evidence indicating that indivduals with MS face higher risk of coronary heart disease. Furthermore, SREBF1 maybe a critical target gene which would significantly contribute to drug development.

**Supplementary Information:**

The online version contains supplementary material available at 10.1186/s41065-025-00388-6.

## Introduction

Cardiovascular diseases rank as the leading cause of death globally, with coronary heart disease (CHD) being a significant factor in serious cardiovascular events [1]. CHD is closely linked to atherosclerosis, which is a chronic inflammatory condition characterized by the involvement of inflammatory mediators such as high-sensitivity C-reactive protein (hs-CRP), interleukin-6 (IL-6), and tumor necrosis factor-alpha (TNF-α). These mediators play a crucial role in the progression of both atherosclerosis and CHD [2, 3]. Research indicates that proactive anti-inflammatory treatments can help prevent CHD and enhance patient outcomes [4]. Beyond traditional risk factors like hypertension, hyperglycemia, body mass index (BMI), and low-density lipoprotein (LDL) levels, various diseases also influence the incidence and progression of CHD. A comprehensive understanding of these intricate interactions is vital for creating effective strategies to alleviate the burden of CHD in affected populations.

MS is an autoimmune disease characterized by inflammation and damage to myelin sheaths in the central nervous system [5]. It predominantly affects young adults, with over 2.8 million individuals affected globally. Studies examining the global disease burden have revealed that the prevalence of MS increased by 10.4% from 1990 to 2016, with variations across different age groups and a particularly significant increase among women compared with men. Recent research highlights a growing trend in the incidence and prevalence of MS, particularly in older populations. A hallmark of MS is the infiltration of lymphocytes into the central nervous system, which triggers neuroinflammation and neurodegeneration [6, 7]. Furthermore, epidemiological studies have demonstrated that individuals with MS face a heightened risk of developing cardiovascular disease (CVD), suggesting a complex interplay between these two health conditions [8, 9]. MS is genetically linked to various cardiovascular risk factors, including triglycerides (TG) and low-density lipoprotein levels, making dyslipidemia a significant biomarker for MS [10, 11]. Recent studies demonstrate that statins are effective in slowing the progression of MS and reducing cognitive decline in patients [12]. It is crucial to accurately assess the risk of atherosclerotic cardiovascular disease to manage patients with MS effectively. Advances in genetic and genomic research, particularly through genome-wide association studies (GWAS), have shed light on the mechanisms connecting these conditions. Nevertheless, further research is necessary to investigate the hereditary links between MS and CHD and to uncover shared pathways that might elevate cardiovascular risk in individuals with MS.

Mendelian randomization (MR) employs genetic variants that are strongly associated with exposure factors as instrumental variables (IVs) to investigate causal relationships between these exposures and outcomes. By utilizing this approach, MR can minimize the influence of common confounding factors, including environmental influences and behavioral aspects [13, 14]. Therefore, the aim of this study is to examine the potential causal relationship between MS and CHD through MR analysis, focusing on how genetic factors may interconnect these two conditions.

## Materials and methods: this study

### Study design

**Fig. 1:**
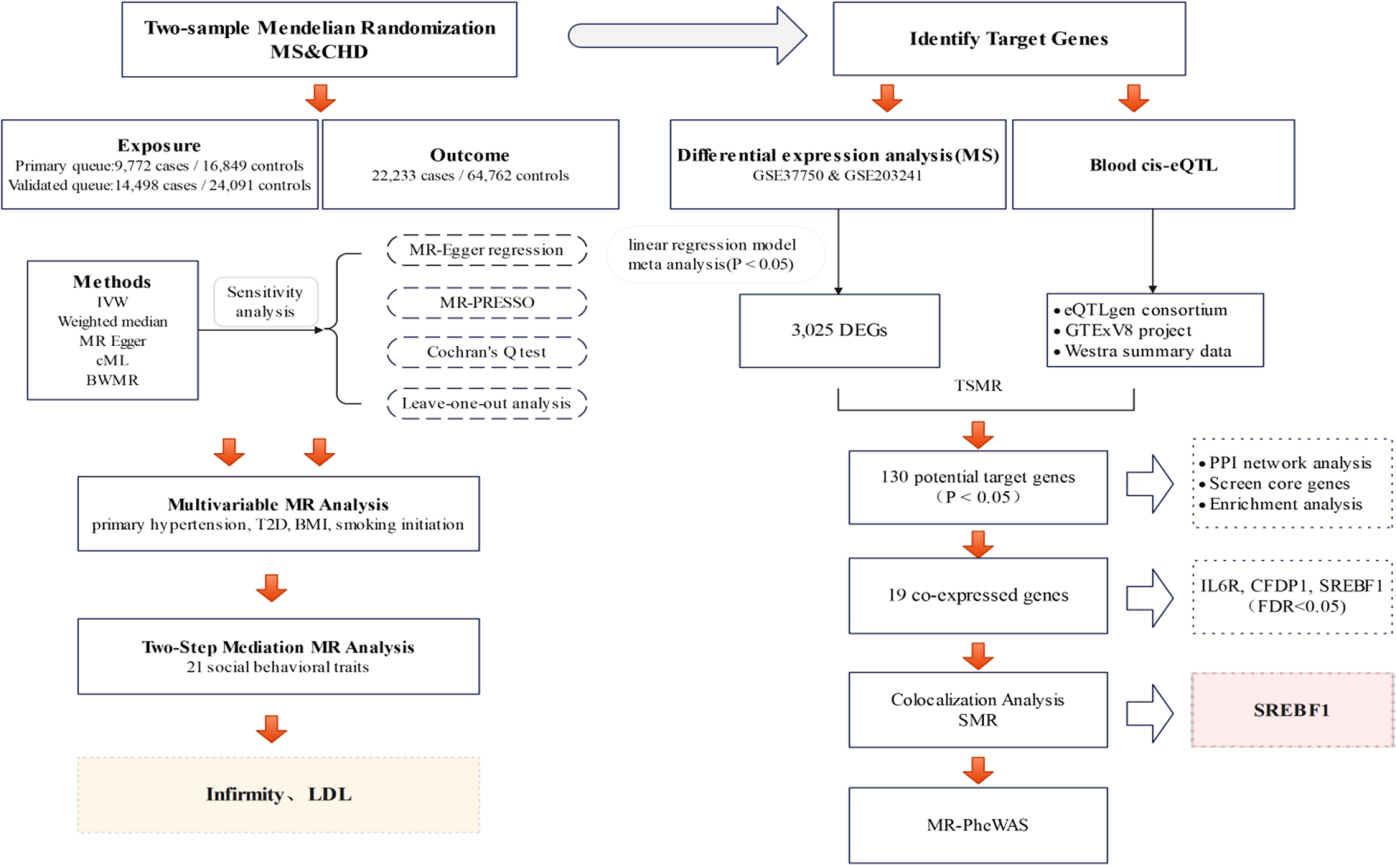
Workflow of the study

This study investigated the causal link between MS and CHD using two-sample Mendelian randomization. To ensure robustness of our findings, we also applied reverse Mendelian randomization, which helps to rule out any potential reverse causation that could skew our results. Additionally, we used multivariate Mendelian randomization (MVMR) to account for confounding factors, thereby clearly identifying the direct impact of MS on CHD. To further understand the role of behavioral traits as mediators, we examined how MS influences these traits and how they, in turn, affect CHD through a two-step Mendelian randomization approach. Moreover, we aimed to pinpoint potential target genes linked to CHD in individuals with MS by screening for differentially expressed genes(DEGs) associated with MS. We then employed the cis-expression quantitative trait loci (cis-eQTL) of these genes as instrumental variables (IVs) to identify genes that were genetically related to the CHD phenotype. To deepen our analysis, we explored the interactions of proteins that regulate these genes using protein interaction networks and evaluated the biological functions of these genes at the cellular level through gene enrichment analysis. Finally, we inferred whether there exists targeted genetic variation between them through colocalization analysis and the summary data-based Mendelian randomization (SMR) method. Finally we examined the possible negative effects of these genes on MS treatment using a phenome-wide MR analysis (Fig. 1).

### Data sources

To estimat causal effects accurately, single-nucleotide polymorphisms (SNPs) were employed as instrumental variables (IVs) in our Meddelian Randomization analysis. For IVs to provide reliable estimates, they must meet three essential criteria: first, they should have a significant association with the exposure; second, they need to be independent of other risk factors that could affect the outcome; and third, their influence on the outcome should occur solely through the exposure, without any direct relationship [15]. We sourced our multiple sclerosis research data from genome-wide association study (GWAS) databases, consisting of 9,772 MS cases and 16,849 controls, which are publicly accessible [16]. To enhance the credibility of our findings, we validated them against a separate cohort consisting of 14,498 MS patients and 24,091 controls [17]. For CHD data, we relied on the CARDIoGRAMC4D consortium, which is a meta-analysis of 22 GWAS studies based on HapMap 2, encompassing 22,233 cases and 64,762 controls [18]. The risk factors for coronary heart disease included primary hypertension, type 2 diabetes, body mass index (BMI), and smoking initiation. Additionally, we determined whether social behavioral traits mediate the relationship between MS and CHD. For this purpose, we selected 21 behavioral traits from the UK Biobank database to evaluate their genetic effects on both MS and CHD and to identify potential mediating variables [19].

### Selection of instrumental variables

We used LDlink [20, 21] to identify and eliminate SNPs that could influence CHD through pleiotropy, thereby minimizing confounding bias. Using the Steiger test [22], we removed SNPs that might cause reverse causation, which strengthened the validity of the instrumental variables (IVs). The selected SNPs were significantly linked to the exposure (P_1_ < 5 × 10^−8^) and were not in linkage disequilibrium (LD, *r*² < 0.001, genetic distance > 10,000 kb). We also filtered out SNPs significantly associated with exposure in the outcome data (P_2_ < 5 × 10^−5^). Furthermore, if there were not enough SNPs, we adjusted the threshold to P^1^ < 1 × 10^−5^. We used the “TwoSampleMR” R package for variable harmonization to identify and exclude palindromic SNPs for which the correct allele direction could not be determined. The F-statistic measures the strength of the association between IV and risk. An F value of less than 10 indicates the potential presence of weak instrumental variables, which may result in biased outcomes. In this study, the *F* value was calculated using the formula F = (β/SE)² (where β is the effect size of the given SNP and SE is the standard error of the effect size) [23].

### Data extraction and ethical statement

To avoid bias related to population differences, we selected all phenotypes from European populations. In addition, we ensured that the summary datasets from the published studies were approved by the relevant institutions. This study utilized R version 4.3.0 (https://www.R-project.org) and the following packages: TwoSampleMR, MendelianRandomization, LDlinkR, MRPRESSO, MVMR, color, Meta, drool, org. Hs.egdb, clusterProfiler, and epichloe packages.

### Statistical analysis

#### Univariate MR analysis

The inverse variance weighted method (IVW)was primarily used in this study for analysis [24]. In addition to this main approach, we also utilized supplementary methods including MR-Egger regression [25], weighted median [26], Bayesian weighted Mendelian randomization (BWMR), which effectively infers causal relationships even in the presence of pleiotropy [27], and constrained maximum likelihood with model averaging (cML-MA), which helps reduce biases arising from both related and unrelated pleiotropy [28]. It was considered statistically significant that a *p*-value of less than 0.05 was achieved.

#### Multivariate MR analysis (MVMR)

This study re-evaluated the direct genetic causal relationship between MS and CHD through MVMR analysis, considering potential risk factors such as primary hypertension, type 2 diabetes, body mass index (BMI), and smoking initiation. Our main analysis used the IVW method, while we also included MR-Egger regression, weighted median regression, and MR-LASSO [29] as supplementary approaches to provide a thorough evaluation. To address weak instrument bias, we calculated the two-sample conditional *F*-value using the MVMR software package. An F statistic exceeding 10 indicates robust instrumental variables, and we made adjustments for the aforementioned confounding factors [30, 31].

#### Two-step mediation MR analysis

This study investigated whether behavioral characteristics served as mediators in the causal relationship between MS and CHD by identifying 21 potential mediators in the mediation Mendelian randomization analysis. Initially, we calculated the total causal effect (β) of the exposure on the outcome using univariate MR analysis. Following this, we used a two-step approach to evaluate the mediating role of social behavioral characteristics. In the first step, we assessed the direct effect (β1) of the exposure on the potential mediator. In the second step, we examined the direct effect (β2) of the potential mediator on the outcome. To determine the indirect effect (or mediation effect), we calculated the product of the coefficients β1 and β2. Ultimately, we divided the mediation effect by the total effect, expressed as [(β1 * β2) / β] to count up the proportion of mediation [32].

#### Sensitivity analysis

This study assessed genetic pleiotropy by using the intercept term from MR-Egger regression, which can introduce bias into the findings. To address this issue, the Mendelian randomization pleiotropy residuals and outliers (MR-PRESSO) test was applied to detect horizontal pleiotropy and outliers [33]. The MR-PRESSO test comprises three components: a global test, an outlier test, and a distortion test. Following the identification of outliers, causal estimates were re-evaluated to strengthen the reliability of the results. Furthermore, the MR-IVW method was employed in conjunction with Cochran’s Q test to assess heterogeneity among single nucleotide polymorphisms (SNPs) [34]. A leave-one-out analysis was also conducted to investigate whether specific SNPs had a significant impact on study outcomes by systematically removing each SNP and recalculating the combined effect of the remaining SNPs.

#### Screening of DEGs and cis-eQTLs

We selected the publicly available transcriptome datasets GSE37750 and GSE203241 from the GEO database (https://www.ncbi.nlm.nih.gov) to study MS [35–37]. The GSE37750 dataset included 8 healthy individuals and 18 MS cases, while the GSE203241 dataset comprised 10 healthy individuals and 16 MS cases. By conducting differential expression analysis on both datasets using linear regression models, adjusting for covariates such as sex and age, we identified standardized differentially expressed genes (DEGs). Following this, we performed a meta-analysis of the results using R software, applying a significance threshold of *P* < 0.05 along with beta coefficients as screening criteria; a beta value > 0 indicated upregulated genes, whereas a beta value 0 indicated downregulated genes.

Using peripheral blood tissue data from the eQTLgen consortium [38], which included 31,684 participants, we investigated the relationship between cis-expression quantitative trait loci (cis-eQTL) from differentially expressed genes in MS and potential target genes linked to CHD phenotypes. To validate our findings, we also utilized whole blood tissue data from the GTExV8 project, comprising 15,201 individuals, along with peripheral blood eQTL summary data from the Westra study, which included 3,511 participants [39, 40]. All SNPs analyzed were required to be located within ± 100 kb of the transcription start site and to have F-values exceeding 10. For genes associated with a single cis-eQTL, we estimated the odds ratio (OR) and confidence interval using the Wald ratio. In cases in which multiple cis-eQTLs were present for a gene, we employed the IVW method to derive effect estimates. Additionally, we implemented false discovery rate (FDR) correction (q < 0.05) to reduce the likelihood of false positives arising from multiple comparisons.

#### PPI network and enrichment analysis

We conducted a protein-protein interaction (PPI) network analysis utilizing the STRING database (https://string-db.org/) to explore the interactions among proteins encoded by the identified genes, applying a minimum interaction score of 0.4 [41]. We also used Cytoscape software to visualize the PPI network (version 3.10.1), and core genes were pinpointed through the degree algorithm available in the cytoHubba plugin [42].

Furthermore, we performed KEGG enrichment analysis to identify genes that are enriched in specific pathways, and we employed Gene Ontology (GO) enrichment analysis to functionally annotate potential target genes across various categories, including biological processes (BP), cellular components (CC), and molecular functions (MF). We established a significance threshold with a *P*-value of less than 0.05 to denote significant enrichment. For visualization, we focused on the top 10 ontologies that exhibited the highest gene enrichment from our analysis [43].

#### Verification of target genes

##### Colocalization analysis

We used Bayesian colocalization analysis to determine whether two phenotypes share causal variants within a specific genomic region. This method employs a Bayesian framework to compute posterior probabilities for five mutually exclusive hypotheses regarding the presence of shared causal variants between the two traits. The hypotheses include: H0, which posits that there are no causal variants for either phenotype; H1, which suggests that causal variants are linked to one phenotype; H2, which indicates that causal variants are associated with the other phenotype; H3, which proposes that two independent SNPs are linked to both phenotypes; and H4, which asserts that one SNP is shared by both phenotypes. Significant colocalization is defined by a posterior probability of H4 (PPH4) greater than 80%, while moderate colocalization is defined by a PPH4 above 50% [44, 45]. Genes that exhibit strong colocalization with CHD may represent potential target genes for further investigation. In this study, we performed color analysis on potential target genes associated with coronary heart disease using a 1000-kb window surrounding each target SNP to enhance the robustness of our findings.

##### SMR method

We evaluated the relationship between potential target genes and MS using the summary data-based MR method (SMR). Additionally, this method was employed within the eQTLGen consortium to identify genetic instruments for target genes using cis-eQTL summary statistics. The HEIDI method was used to test the findings, where a *P*-value of less than 0.05 suggests significant heterogeneity in the results [46, 47].

##### Phe-WAS

To explore the potential side effects of target genes, we utilized a dataset of 783 diseases [48] from the UK Biobank cohort. Subsequently, we established uniform parameters based on the cis-eQTL of target genes found in blood samples and conducted MR analysis using the MR-IVW method. Causal effects were deemed statistically significant if the FDR was less than 0.05.

## Results

### Univariate MR analysis

**Fig. 2:**
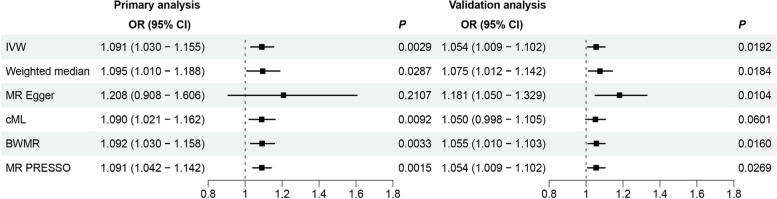
Associations between genetically predicted MS and CHD in the primary and validation analyses. BWMR: Bayesian weighted Mendelian randomization; cML: constrained maximum likelihood with model averaging

In this study, all SNPs included in the final analysis exhibited *F*-values exceeding 10 (Supplementary Tables 1–2). The findings suggest that a higher genetic susceptibility to MS may increase the risk of CHD, as shown by the following odds ratios: IVW OR = 1.091 (95% CI: 1.030, 1.155, *P* = 0.0029), WM OR = 1.095 (95% CI: 1.010, 1.188, *P* = 0.0287), and BWMR OR = 1.092 (95% CI: 1.030, 1.158, *P* = 0.0033). Although the MR Egger analysis indicated a negative result, the complementary method of cML-MA provided evidence of a positive association, yielding an odds ratio of 1.090 (95% CI: 1.021, 1.162, *P* = 0.0092). Furthermore, the MR-PRESSO test demonstrated a positive correlation between MS and CHD, with an OR of 1.091 (95% CI: 1.042, 1.142, *P* = 0.0015), which aligns with the estimates obtained from the IVW method. Additionally, a positive causal relationship was confirmed in the replication cohort(IVW OR = 1.054;95% CI: 1.009, 1.102;*P* = 0.0192)), reinforcing the robustness of this findings (Fig. 2). Finally, the association remained significant in the meta-analysis results, with an OR of 1.068 (95% CI: 1.031, 1.106, *P* = 0.0002) (Supplementary Fig. 1).

We conducted a reverse Mendelian randomization analysis using CHD as the exposure and MS as the outcome, with IVW as the primary causal estimate (Supplementary Table 3). The results did not indicate a causal relationship, which helps to rule out the possibility of reverse causality.

### Sensitivity analysis

The MR-Egger regression revealed an intercept term close to zero and a *P*-value greater than 0.05, which indicates that the likelihood of pleiotropy is low. Furthermore, no evidence of pleiotropy was revealed in the MR-PRESSO global test. In the heterogeneity assessment, the Q statistic was 11.846, accompanied by a *P*-value of 0.855, which indicated that there was no significant heterogeneity in the MR results. Within the replication cohort, we discovered an outlier (rs3130283) that influenced the findings. Nevertheless, even after excluding this outlier, the MR-PRESSO results remained consistent (Supplementary Table 4). By employing the “leave-one-out” method, where individual SNPs were removed one at a time, the IVW results for the remaining SNPs closely mirrored the total effect value including all SNPs, and we found no SNPs to have a significant impact on the outcome, thereby confirming the stability of the MR results (Supplementary Fig. 2).

### MVMR analysis

**Fig. 3:**
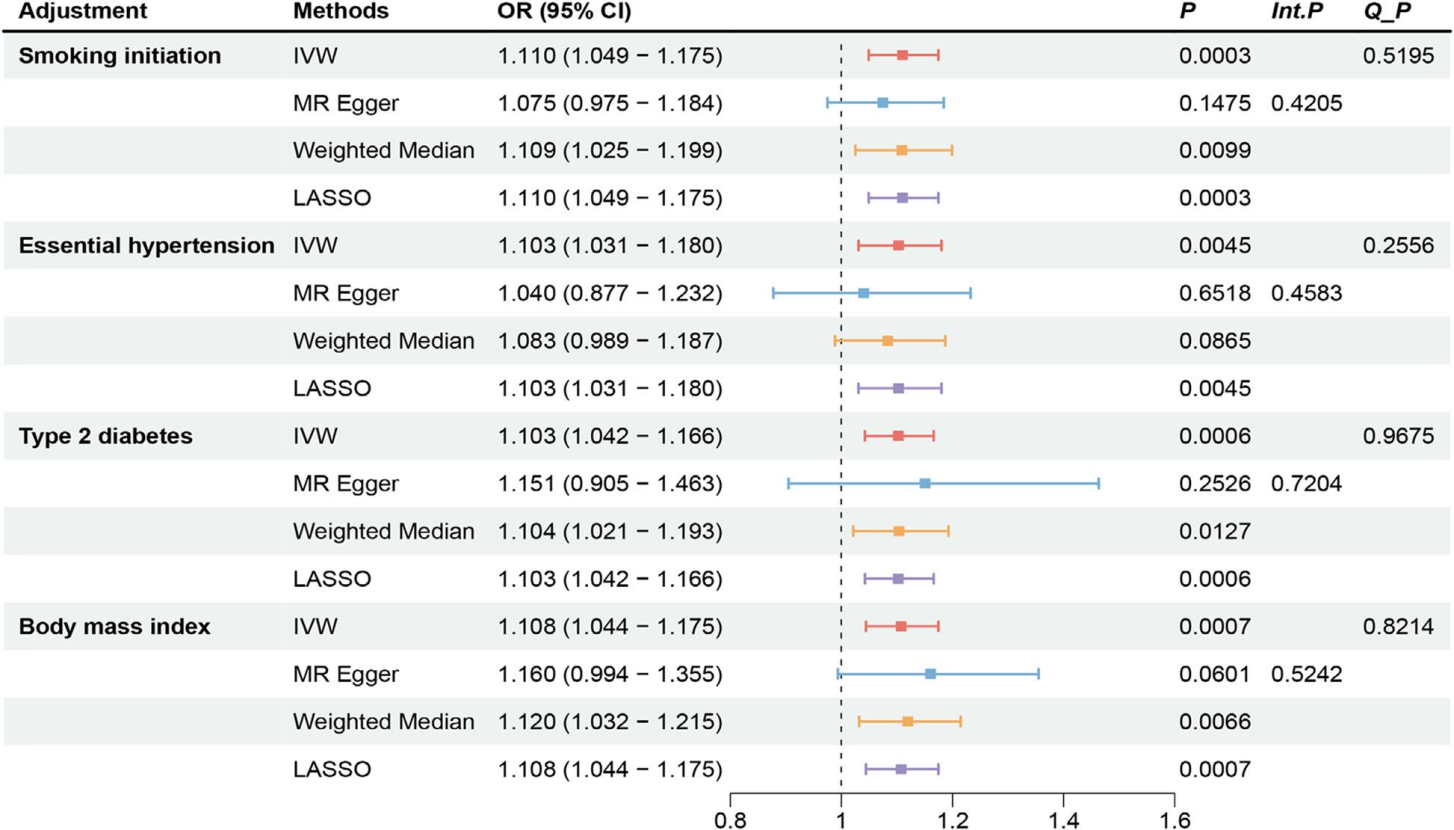
Multivariate MR analysis. We re-evaluated the genetic links between MS and CHD, considering four confounding factors: Essential hypertension, Type 2 diabetes, Boss mass index, and Smoking initiation. Int.P : MR Egger intercept; Q_P: Cochran’s Q test

This study aimed to evaluate the direct effect of MS on coronary heart disease by controlling for factors such as primary hypertension, type 2 diabetes, BMI, and smoking initiation using multivariable Mendelian randomization (MVMR) analysis. After adjusting for the four confounding factors, we found that the positive causal association remained significant. This indicates that the effect of MS on CHD is independent of the risk factors. Sensitivity analysis revealed that neither the MR-Egger regression nor the IVW methods showed evidence of pleiotropy or heterogeneity. Using the supplementary method of MR-LASSO, the estimated results were consistent with those from the IVW analysis, suggesting stability (Fig. 3). All F-values for the exposure phenotypes in this study were greater than 10, indicating a low likelihood of instrument bias.

### The relationship between social behavioral characteristics to MS and CHD

When these characteristics are considered as exposure factors, we found that higher BMI, LDL, and systolic blood pressure levels are associated with an increased risk of CHD, loneliness and frailty also showed a positive genetic correlation with CHD; however, insomnia shows a negative genetic correlation with CHD. After correction for FDR, all other characteristics remained significantly associated with the causal relationship to CHD (FDR < 0.05), except for systolic blood pressure. Additionally, we only identified LDL levels as a risk factor for MS (Supplementary Tables 5–6).

 To further screen for mediating factors, a two-step mediation MR analysis. Surprisingly, frailty has a mediating effect in the genetic causal relationship between MS and CHD, with a mediation effect proportion of 20.2% (*P* = 0.026). Furthermore, an interesting phenomenon where LDL may be a common risk factor for both MS and CHD was observed by us ; the mediation MR analysis found that MS may mediate 11.2% of the genetic susceptibility of LDL to CHD (Table 1).

**Table 1:** Effect of infirmity and LDL in the association between MS and CHD. TE: Total effect; DE: Direct effect; IE: Intermediary effect; Infirmity: Long-standing illness, disability or infirmity

Exposure/Outcome	Mediator	TE		DE		IE		*p*val	Proportion
		β	OR (95%CI)	β	OR (95%CI)	β	OR (95%CI)		
MS/CHD	Infirmity	0.096	1.101(1.047,1.158)	0.077	1.080 (1.024,1.139)	0.019	1.020 (1.002,1.038)	0.026	20.2%
LDL/CHD	MS	0.539	1.714(1.335,2.201)	0.479	1.614(1.249,2.087)	0.06	1.062(1.001.1.127)	0.042	11.2%

## Target gene

### Screening genes associated with CHD using cis-eQTL as an instrumental variable

To explore the genetic underpinnings of MS, we conducted a meta-analysis of DEGs sourced from the GSE37750 and GSE203241 datasets. This analysis identified 3,025 DEGs, with a significance threshold of *P* < 0.05. Among these, we identified 1,509 upregulated and 1,516 downregulated genes in patients diagnosed with MS (Supplementary Table 7).

**Fig. 4:**
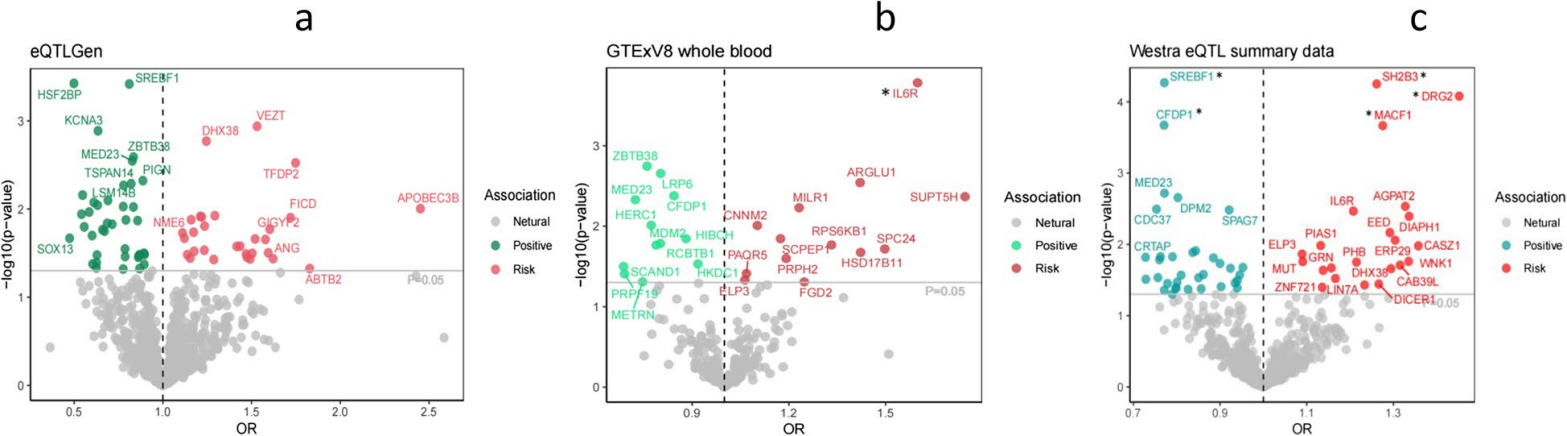
We analyzed SNPs linked to blood cis-eQTLs from three data sources to identify differentially expressed genes associated with MS,and conducted MR analysis to find potential target genes for CHD. **a **eQTLGen consortium; **b **GTExV8 whool blood issue; **c **Westra study peripheral blood. *FDR<0.05

In the eQTLGen discovery cohort, we identified 71 genes that were causally associated with coronary heart disease using DEGs and the two-sample Mendelian randomization method. In the subsequent replication cohort, we identified 25 potential target genes in GTExV8 whole blood tissue and 61 potential target genes in Westra eQTL summary data from peripheral blood, with a significance threshold of *P* < 0.05. Notably, among the identified genes, IL6R, SH2B3, CFDP1, SREBF1, DRG2, and MACF1 remained significant even after applying FDR correction, with a q-value of less than 0.05 (Fig. 4).

### PPI and enrichment analysis

**Fig. 5:**
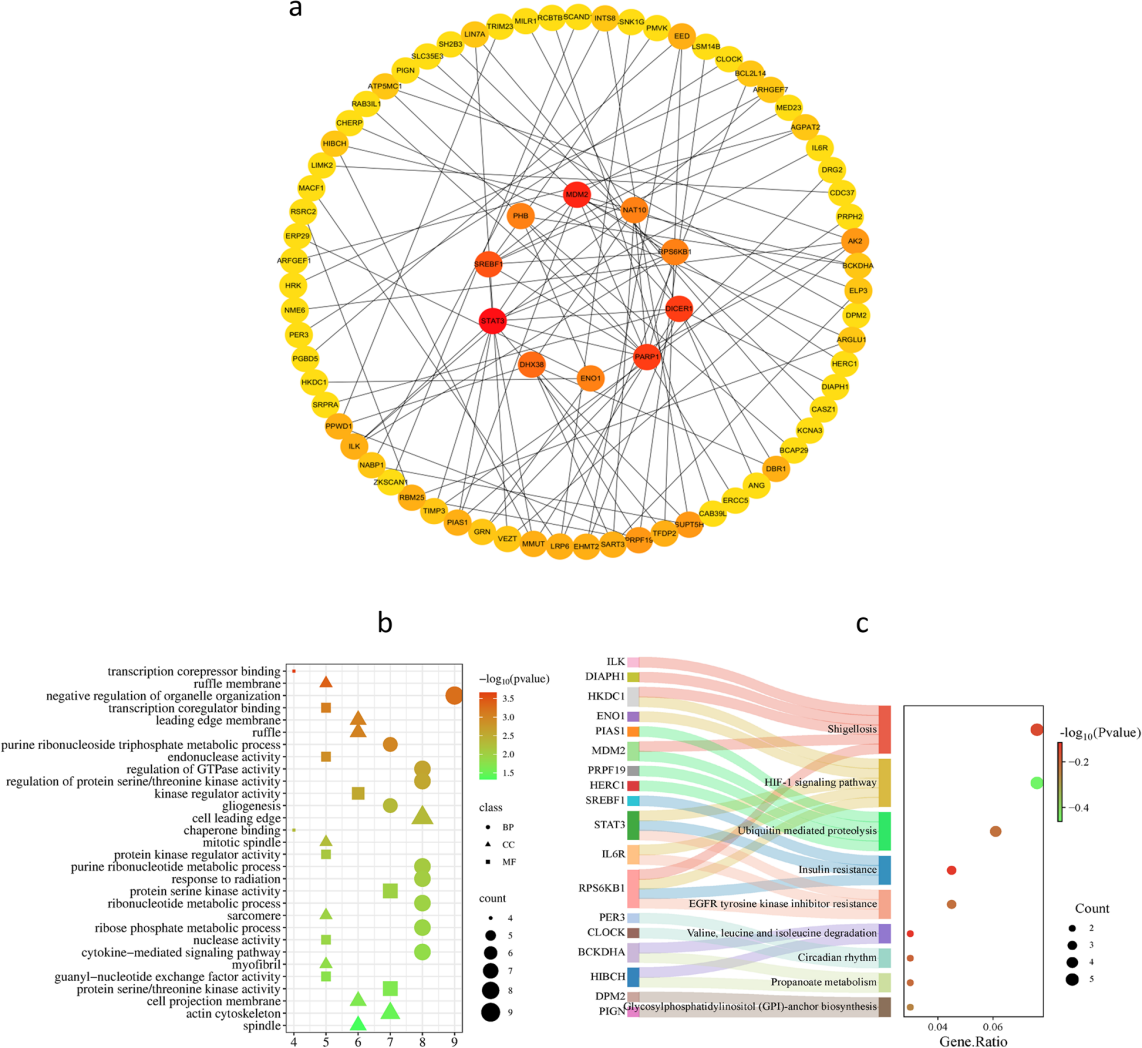
**a** protein-protein interaction (PPI) network analysis. We identified 77 genes with potential effects through the protein interaction network, where darker colors signify stronger effects. From these, we selected the top 10 genes based on their scores. **b** GO enrichment assays show the functions of potential target genes, which include biological processes (BP), cellular components (CC), and molecular functions (MF), with the symbol size indicating the level of enrichment. Bubble chart: the x-axis indicates the number of enriched genes, while the y-axis represents their corresponding functions. **c** This study utilized KEGG enrichment analysis to identify genes associated with specific pathways. Sankey-dot plot: the horizontal axis illustrates the proportion of enriched genes, and the vertical axis illustrates the corresponding pathways of enrichment

We conducted a protein interaction network analysis focusing on proteins encoded by 130 genes, which were selected from three different data sources due to their potential genetic links to CHD. Our findings indicate that 77 of these genes are likely interconnected. Through network topology analysis, we pinpointed the top 10 core genes: MDM2, STAT3, SREBF1, RARP1, DICER1, DHX38, PHB, RPS6KB1, ENO1, and NAT10, suggesting that these genes may have significant associations with CHD (Fig. 5a).

To elucidate the potential roles of these genes, we performed an enrichment analysis. The results of our Gene Ontology (GO) enrichment analysis indicated that the enriched genes were mainly involved in the ribonucleotide metabolic process and played a negative role in organelle organization. In terms of cellular components, we found significant enrichment in the cell front region and the actin cytoskeleton. When examining molecular functions, protein serine/threonine kinase and kinase regulator activities emerged as the most significant. Additionally, our pathway enrichment analysis revealed that these genes predominantly influenced the hypoxia-inducible factor-1(HIF-1) signaling pathway, ubiquitin-mediated proteolysis, and insulin resistance (Fig. 5b and c).

### Colocalization analysis

**Fig. 6:**
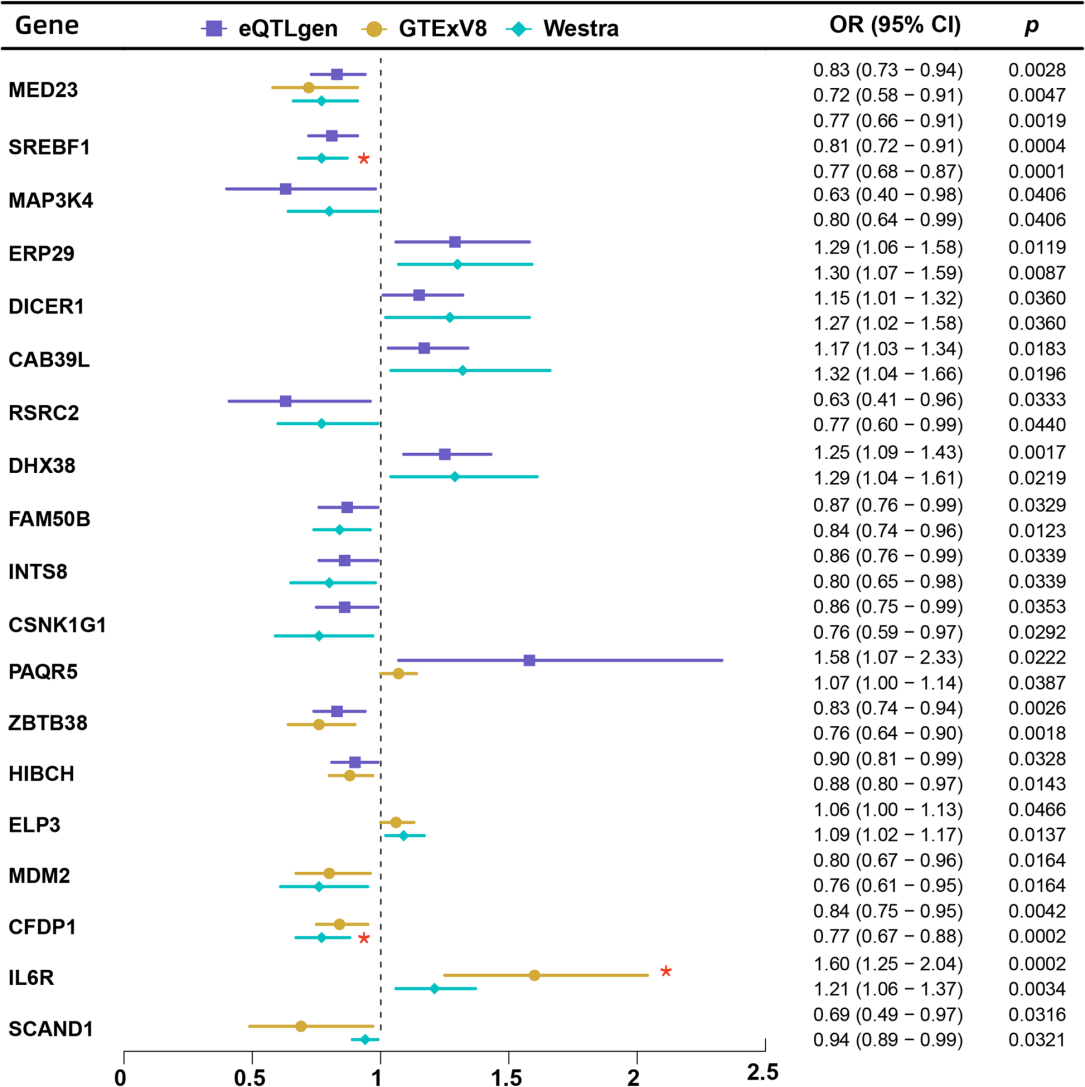
Our study selected 19 genes that were co-expressed in at least two different datasets, showing consistent effects across these datasets.*FDR<0.05

To enhance the reliability of our study results, we selected genes coexpressed in at least two different data sources that demonstrated consistent effects across these sources. Our findings indicated that MED23 exhibited a negative genetic association with CHD in three distinct datasets. Additionally, we identified a total of 18 present genes in two of the data sources. Of these, 11 genes were found to have a negative correlation with CHD, including SREBF1, MAP3K4, RSRC2, FAM50B, INTS8, CSNK1G1, ZBTB38, HIBCH, MDM2, CFDP1, and SCAND1. Conversely, the remaining 7 genes—ERP29, DICER1, CAB39L, DHX38, PAQR5, ELP3, and IL6R—were associated with an increased risk of developing CHD (Fig. 6).

**Fig. 7:**
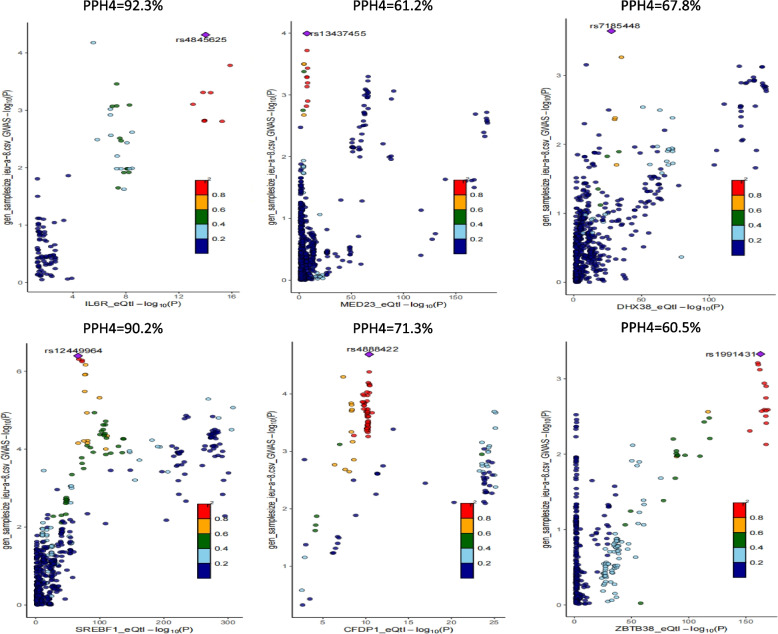
Colocalization Analysis.We offered a detailed comparison of genetic loci associated with the cis-eQTL and CHD genes.SREBF1 and IL6R were genetically linked to CHD, showing high PPH4 of 90.2% and 92.3%; MED23, DHX38, ZBTB38, and CFDP1 exhibited moderate co-localization with CHD (PPH4 >50%)

We carried out an enrichment analysis so as to elucidate what potential roles these genes act. The findings revealed that SREBF1 and IL6R are genetically linked to CHD, with high PPH4 levels of 90.2% and 92.3%, respectively. Furthermore, we observed that MED23, DHX38, ZBTB38, and CFDP1 exhibited moderate co-localization with CHD, with their PPH4 falling between 50% and 80% (Fig. 7).

### Summary data-based mendelian randomization (SMR) analysis

We focused on the core gene SREBF1, which demonstrated positive colocalization, and conducted an SMR analysis in the context of MS. What was revealed by our findings is a negative genetic association between SREBF1 expression and CHD, supported by statistical values of P_SMR_=0.0218 and P_HEIDI_=0.2806, with the most significant SNP identified as rs12951376 (Supplementary Table 8). Additionally, we observed that SREBF1 is significantly downregulated in patients with MS, highlighting its potential as a crucial gene for treating MS patients who are also affected by CHD.

### Phenome-wide MR analysis

**Fig. 8:**
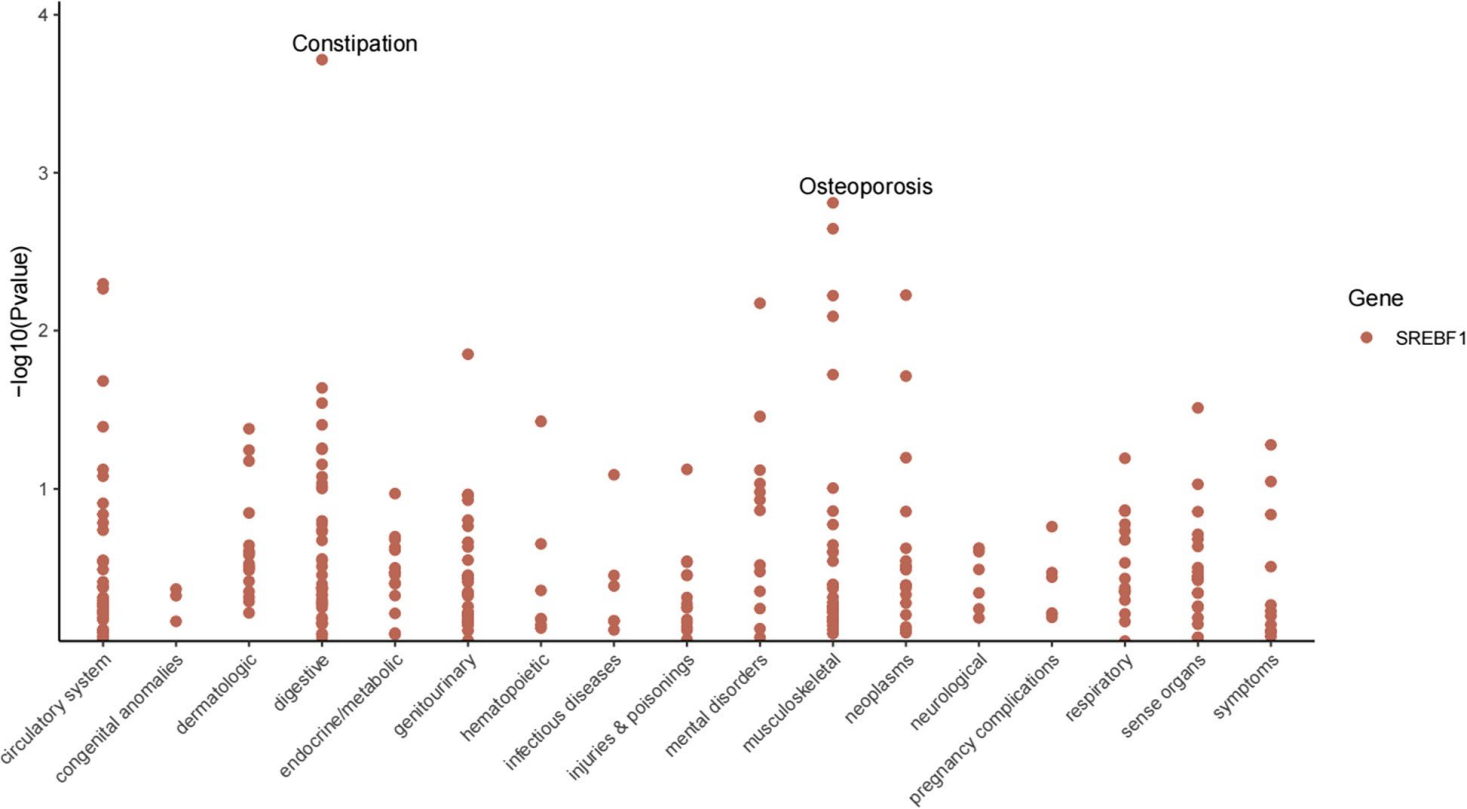
Manhattan plot for phenome-wide MR results of blood SREBF1.The vertical axis represents the *P*-values of the phenome-wide MR results, the horizontal axis represents disease categories, and each point represents a disease trait

We conducted extensive MR screening for 783 diseases or traits and found no significant association between SREBF1 and diseases excluding MS and CHD (FDR > 0.05) (Supplementary Table 9). We observed some trends; for instance, higher levels of SREBF1 expression in the blood may increase the risk of constipation[OR = 1.220, 95%CI(1.099, 1.135),* P* = 0.0002], additionally, high SREBF1 expression may also increase genetic susceptibility to osteoporosis[OR = 1.250, 95%CI(1.089, 1.434),*P* = 0.0016] (Fig. 8).

## Discussion

Patients with multiple sclerosis (MS) have an increased incidence of cardiovascular diseases, but genetic research linking MS to coronary heart disease (CHD) remains scarce. Our study investigated the causal relationship between MS and CHD, thus filling a critical gap in the existing literature. Using advanced statistical techniques, we explored the direct impact of MS on CHD risk. Our findings revealed a significant association between genetic susceptibility to MS and an increased risk of developing CHD, suggesting that MS may serve as an independent risk factor for cardiovascular diseases. This finding aligns with existing literature that highlights the cardiovascular risks associated with autoimmune conditions [49, 50], emphasizing the need for targeted interventions to mitigate these risks.

MS is a serious neurological disease that worsens over time, leading to declining health and significant physical disabilities. Our assessment found the relationship between various common social behavior characteristics and MS, revealing that MS increased the risk of infirmity, which also increased the genetic susceptibility to CHD. This long-standing illness state often indicates poorer clinical outcomes [51]. As the population ages, frailty is increasingly associated with higher rates of cardiovascular disease (CVD), indicating a potential bidirectional relationship. Moreover, frailty has emerged as an independent prognostic factor for cardiovascular events, with improvements in frailty status linked to a reduced risk of developing cardiovascular diseases [52, 53]. Compared with healthy individuals, in the case of MS, a debilitating neurological disorder, the risk of experiencing frailty is roughly 15 times greater [54]. Our study employed mediation Mendelian randomization analysis, demonstrating that frailty serves as a vital mediator, accounting for 20.2% of the genetic causal relationship between MS and CHD, which underscores the importance of frailty as a potential biomarker for assessing cardiovascular risk in patients with MS. Consequently, addressing frailty may help mitigate the heightened incidence of CHD among those with MS. These findings open the door to targeted intervention strategies aimed at improving overall health and quality of life for patients with MS and CHD, ultimately contributing to a decrease in adverse clinical outcomes.

Identifying target genes is essential for disease diagnosis and treatment. In this study, we identified 19 potential target genes that are relevant to CHD treatment in patients with MS. These genes were identified using three distinct data sources. Notably, SREBF1 exhibited negative regulatory effects on both MS and CHD, indicating its potential role as a regulator of both conditions. Gene enrichment analysis revealed that SREBF1 is primarily involved in transcription regulation and nucleic acid metabolism and may also play a role in insulin resistance. These findings provide valuable insights into new treatment options for CHD in patients with MS and highlight SREBF1 as a significant gene of interest. Additionally, phenome-wide MR pointed out beneficial uses for therapeutics that target the gene and mentioned some possible safety concerns.

In the context of our study, it is essential to highlight the pivotal role of the SREBF1 gene in mediating the relationship between MS and CHD. SREBF1(Sterol Regulatory Element Binding Transcription Factor 1) is crucial in regulating the expression of key genes involved in cholesterol metabolism, including LDLR and HMGCR [55, 56]. As a transcription factor, SREBF1 plays a vital role in maintaining lipid homeostasis, which is not only significant for metabolic processes but also holds importance in tumor growth regulation [57, 58]. Furthermore, It is implicated in the modulation of lipid metabolism and may participate in the hydrolysis of cholesterol esters through autophagy regulation [59]. The connection between SREBF1 and diseases such as MS and CHD is underscored by its regulatory influence on cholesterol uptake and synthesis.

Elevated LDL-C levels are a classical marker of cardiovascular risk, with high concentrations contributing to the development of atherosclerosis. Previous research has indicated that LDL can infiltrate MS plaques, indicating a pathological crossover between these conditions [60]. Moreover, the oxidative products of cholesterol have pro-inflammatory and neurotoxic effects, which are linked to demyelination associated with adverse outcomes in MS [61, 62]. Through our Mendelian randomization analysis, we found that increased LDL is associated with a higher genetic susceptibility to both MS and CHD, and we propose that MS might play the role of a potential mediator in the relationship between LDL and CHD. Thus, LDL’s role as a biomarker for MS is not just incidental; it has significant implications in understanding disease mechanisms and developing potential therapeutic avenues. The SREBF pathway is crucial for cholesterol metabolism, and exciting recent findings suggest that SREBP activation can lead to the activation of inflammatory caspases, thereby intensifying inflammatory responses and promoting the development of autoimmune diseases [63–65]. This expanding understanding positions SREBF1 as not only a mediator of lipid homeostasis but also an influencer of autoimmune disease pathophysiology.

Intriguingly, our study revealed that SREBF1 expression was significantly downregulated in patients with MS. This downregulation intersects with its negative regulatory effects on both MS and CHD, indicating that LDL acts as a downstream factor regulated by SREBF1, contributing to the progression of these diseases. Addressing the relatively sparse research on SREBF1 in patients with MS presents a compelling avenue for future investigations. Given its role as a potential therapeutic target, a deeper exploration of SREBF1 may reveal novel strategies targeting mitigating the consequences of MS and related cardiovascular impairments. Understanding the intricate balance that SREBF1 maintains in lipid metabolism and inflammation could lead to innovative approaches to managing these interconnected health issues. In addition, our results shown that the upregulation of SREBF1 may pose risks for gastrointestinal dysfunction and osteoporosis, which could be a consideration when targeting SREBF1 for the treatment of CHD and MS in the future.

### Limitation

We admit that there are several limitations that must be addressed in this study when interpreting the findings. First, the sample size may not fully capture the heterogeneity of the multiple sclerosis (MS) population, potentially limiting the generalizability of the results. Additionally, the absence of laboratory validation for the identified genes restricts the confirmation of their functional roles in the pathophysiological mechanisms linking MS and coronary heart disease (CHD). Furthermore, reliance on existing datasets may introduce confounding factors due to batch effects or variations in data collection methodologies. These limitations highlight the necessity for caution when extrapolating the results to broader clinical contexts. Future investigations should incorporate experimental methodologies and diverse cohorts to validate the findings and enhance their applicability.

## Conclusion

In conclusion, this study established a significant causal relationship between multiple sclerosis and coronary heart disease, highlighting the role of potential mediators and target genes. The identification of these genetic associations not only contributes to a deeper understanding of cardiovascular risk in patients with MS but also opens avenues for novel therapeutic strategies. By elucidating the underlying mechanisms, these findings can pave the way for targeted interventions to mitigate cardiovascular complications in this vulnerable population. Further studies are warranted to explore these pathways and validate the clinical implications of the identified genetic factors, ultimately contributing to improved management of patients with coexisting MS and CHD.

## Supplementary Information

Supplementary Material 1.

## Data Availability

Data is provided within the manuscript or supplementary information files.
