# Supplementary figures and images for: SREBF1, a target gene of multiple sclerosis and coronary heart disease: based on mendelian randomization study
Source: Hereditas. 2025 Feb 14;162:22. doi: 10.1186/s41065-025-00388-6 (PMC11827142; doi:10.1186/s41065-025-00388-6)

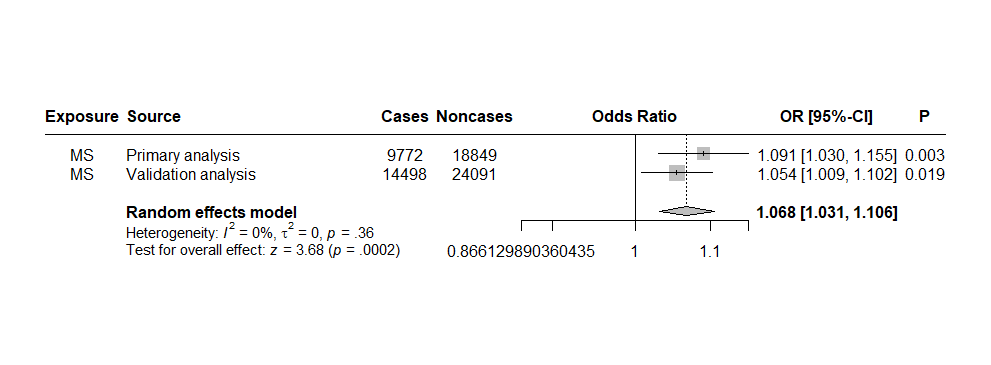

Supplement: Supplementary file 1 — Supplementary Material 1. [file 41065_2025_388_MOESM1_ESM.zip › Supplementary Materials/Supplementary Figure1.png]

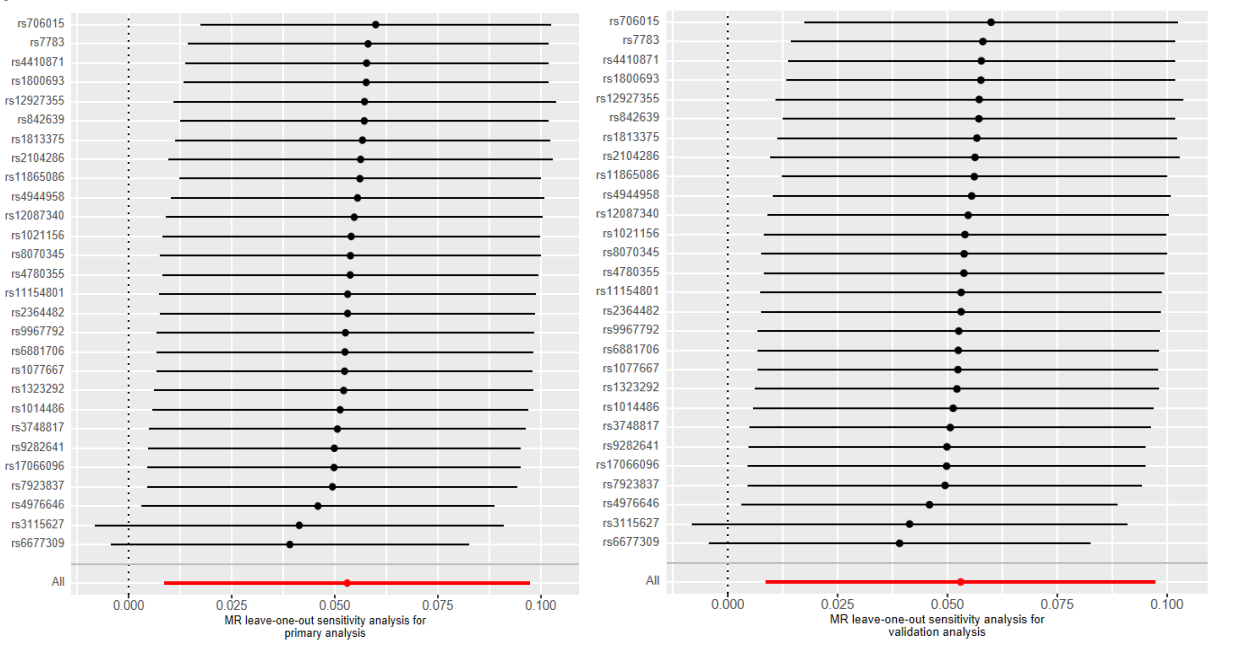

Supplement: Supplementary file 1 — Supplementary Material 1. [file 41065_2025_388_MOESM1_ESM.zip › Supplementary Materials/Supplementary Figure2.png]
